# School Social Fragmentation, Economic Deprivation and Social Cohesion and Adolescent Physical Inactivity: A Longitudinal Study

**DOI:** 10.1371/journal.pone.0099154

**Published:** 2014-06-16

**Authors:** Roman Pabayo, Michel Janosz, Sherri Bisset, Ichiro Kawachi

**Affiliations:** 1 Department of Social and Behavioral Sciences, Harvard School of Public Health, Boston, Massachusetts, United States of America; 2 École de Psychoéducation, Université de Montréal, Montréal, Québec, Canada; 3 School Environment Research Group (SERG), Université de Montréal, Montréal, Québec, Canada; 4 Université de Montréal Public Health Research Institute (IRSPUM), Montréal, Québec, Canada; 5 Plateforme d'évaluation en prévention de l'obésité, Centre de recherche, institut universitaire de cardiologie et de pneumologie de Québec (CRIUCPQ), Université de Laval, Québec City, Québec, Canada; Pennington Biomedical Research Center, United States of America

## Abstract

**Objectives:**

To examine the independent influence of school economic deprivation, social fragmentation, and social cohesion on the likelihood of participating in no physical activity among students.

**Methods:**

Data are from a large-scale longitudinal study of schools based in disadvantaged communities in Quebec, Canada. Questionnaires were administered every year between 2002 and 2008 among n = 14,924 students aged 12 to 18 from a sample of 70 schools. Cross-sectional and longitudinal analyses were conducted. Multilevel modeling was utilized to account for the clustering of students within schools. Schools were categorized as being low, moderate or high economic deprivation, social fragmentation and social cohesion. Those who indicated that they do no participate in any physical activity during the week were identified as being physically inactive.

**Results:**

In baseline multilevel cross-sectional analyses, adolescents attending schools in the highest (compared to the lowest) levels of socioeconomic deprivation and social fragmentation were more likely to be physically inactive (OR = 1.33, 95% CI = 1.03, 1.72; and OR = 1.24, 95% CI = 0.98, 1.56, respectively). Conversely, students attending schools with the highest cohesion were less likely to be physically inactive (OR = 0.78, 95% CI = 0.61, 0.99). In longitudinal analysis, physically active students who attended schools with the highest social fragmentation were more likely to become physically inactive over two years (OR = 1.65, 95% CI = 1.09, 2.51).

**Conclusion:**

The school socioeconomic environment appears to be an important contextual influence on participation in no physical activity among adolescents. Following adolescents beyond two years is necessary to determine if these environments have a lasting effect on physical activity behavior.

## Introduction

Physical activity is important to the growth and development of children and adolescents [1]. Recommendations of one hour a day of Moderate to Vigorous Physical Activity (MVPA) have been made [2], [3] for optimal health [1]. However, recent findings among Canadian youth indicate only 9% and 4% of boys and girls respectively meet these recommendations [2]. In 2011, in the United States, 13.8% of American adolescents reported that they had not participated in at least 60 minutes of MVPA in the previous 7 days [4]. Furthermore, as children age into adolescence, physical activity, on average declines [5], [6], [7]. This highlights the need to gain a better understanding of factors that may influence physical inactivity.

While family practices are known to be key determinants [8], [9], growing evidence suggests that physical activity is associated with conditions in the wider environment, including neighborhood socioeconomic conditions, deprivation, and social disorganization [10], [11], [12]. School environments may be influential on students' behaviors because adolescents spend most of their waking hours within schools [13], [14]. Indeed, the importance of the school as an influential institution on students' behaviors has been argued theoretically [15] and demonstrated empirically [14], [16], [17], [18], [19], [20].

For example, members of the school environment, such as teachers and staff can influence adolescent behavior. Students who perceive to receive encouragement from their teachers are more physically active [21], [22]. School climate measures such as safety and feelings of belonging have been positively associated with physical activity [23], [24].

Area-level socio-economic characteristics such as the socio-economic status of the neighborhood have shown to be significant for moderate to vigorous physical activity. Neighborhood economic deprivation, social fragmentation, and social cohesion are three common characteristics that have been studied [24]–[29]. There is a potential opportunity to include these three socioeconomic characteristics in the same investigation in the school setting.

School-level economic deprivation is a collective measure of average SES of student populations. The greater the level of economic deprivation, the more disadvantaged the student population within a school. Children from disadvantaged backgrounds, such as those from low-income households [25],[26] participate in less physical activity. Thus, adolescents from deprived backgrounds may have limited access to resources and facilities that are needed to promote physical activity.

There are two potential mechanisms whereby the economic deprivation contextual effect may influence student physical activity. First, resources, such as equipment and school staff may be less available in socioeconomically disadvantaged schools [27], [28]. Secondly, peers, teachers, and staff may also influence students' behavior [29], [30]. In particular, low levels of physical activity among peers (due to clustering of lower SES children in some schools), may adversely affect an individual student's propensity to exercise – a type of social contagion effect [29], [30]. In other words, physical activity behaviors of those around students might encourage others to be physically active.

A dimension of the socioeconomic environment that is conceptually distinct from deprivation is “social fragmentation”, which relates to instability in social relationships (e.g. captured by rapid population turnover). Social fragmentation was originally created to measure the non-economic deprivation aspects of areas, and was later described as the level of social integration and social support attributed to ties within a community [31]. Recently, social fragmentation has been theorized as the opposite of social cohesion or integration [32], [33]. Previous research has investigated the relationship between residential neighborhood social fragmentation and physical activity [10], [34] and mental health outcomes [33], [35]. However, the concept of social fragmentation captures an important yet often overlooked characteristic of the school environment. Previous research has investigated the role of school social fragmentation on psychotic disorders [36], but its role on physical activity has not yet been explored. School-level social fragmentation could influence physical activity through several mechanisms. High stability within a school, characterized by low teacher and student turnover, may promote more durable social ties between students (as well as between teachers and students), which facilitate greater investment by teachers to create and maintain extracurricular activities (ECAs) and other opportunities for students to become active. Stronger ties can also lead to increased student school spirit or morale, which may lead to greater participation in ECAs.

“School climate” has been defined by how harmoniously students, teachers, principals and other staff relate to each other within a school. Instruments to measure school climate encompass both the frequency and closeness of social interactions, as well as broader dimensions such as perceived justice and equity [37], [38]. In other words, there is substantial overlap between school climate and the construct of social cohesion, which has been extensively examined within the neighborhood effects literature [39]. Students attending schools with higher levels of cohesion (stronger “school climate”) may be more likely to be physically active because they have a more positive perception of their school, enjoy spending time with other students, which combined can lead to greater participation in school activities. Likewise, teachers who enjoy spending time with other students and teachers may be more likely to initiate or support school activities.

Although school-level economic deprivation, social fragmentation, and social cohesion could be seen as conceptually different, it could be argued that these factors could be in fact related. For example, economically deprived school populations might also have high teacher turnover and student dropout rates. Similarly, students attending socially fragmented schools might feel less connected with their fellow peers, teachers, and schools. Investigations should include determining that the three socioeconomic factors are distinct. Nonetheless, previous work that investigated the role of socioeconomic factors on health outcomes has included economic deprivation, social fragmentation, and social cohesion.

The role of the socio-economic environment of the school on physical inactivity has not been thoroughly examined; an investigation of the possible additive effect of school-level economic deprivation, social fragmentation, and social cohesion on this behavior is warranted. Therefore, the goal of this investigation was to examine whether these school-level factors are independent predictors of participating in no physical activity among adolescents participating in a large-scale longitudinal study in the province of Quebec Canada.

## Methods

### Ethics Statement

Ethical approval was provided by the Université de Montréal Institutional Review Board. For each student, investigators obtained written consent from his or her parents or guardians. Data for this study is available on request by contacting the Groupe de recherche sur les environnements scolares (GRES)/School Environment Research Group.

Participants (n = 14,924) are from the New Approaches New Solutions (NANS) longitudinal data set (2002–2008). 70 schools were selected through a stratified random sampling procedure to represent the 200 secondary schools located in disadvantaged communities in Quebec in terms of geographical location size, and language. An additional 10 schools from communities of average socioeconomic level were also randomly selected to provide variability in SES at the school level. The total sample comprises 60 French-speaking schools, 12 small schools (199 students or less) 36 mid-size schools (200 to 999 students), and 23 large schools (1000 students or more). Self-reported questionnaires were administered to students annually. For this study, data that were gathered during the 2006/07 (baseline) and 2007/2008 (follow-up) school years were utilized. At baseline, children were aged 12-7 years. Those lost to follow-up were more likely to be male, Non-European immigrant; older and reported significantly lower individual social cohesion scores. All surveys were administered in class by teachers supervised by trained researchers.

### Measures

#### Dependent Variable

At baseline and follow-up, students reported their physical activity by completing a self-administered questionnaire, which asked, “Altogether, how many hours a week do you spend doing physical activities?” Response options included: I don't do any, 1 to 2 hours, 3–5 hours, 6–8 hours, 9 to 11 hours, or 12 or more hours. Responses were dichotomized to physically inactive (I don't do any) versus physically active (all remaining categories). We chose to dichotomize the outcome in this manner in order to minimize misclassification. For example, by dichotomizing students' physical activity responses into no physical activity and any physical activity, we are correctly identifying those students who are physically inactive.

Covariates included individual, familial, and school-level characteristics measured at baseline. Individual variables included the student's sex, age, and Immigrant status (Canadian-born, Aboriginal, Non-European, and European). Familial variables included family status (Single parent or more than 1 parent) and familial adversity. An index of risk for familial adversity was developed using nine indicators; wealth, home educational resources, mother and father's education, occupation and marital status of the parents, number of times the family has moved, and sibling school dropout. A score of 1 to 9 was produced; a higher score indicated a greater risk for adversity. Participants were categorized into having low risk (1 to 3), moderate (4 to 6) and high (7 to 9).

### Covariates measured among students attending French Schools

Participants were asked “Since the beginning of the school year, have you gotten involved in ECA's organized by the school or in cooperation with the school (e.g. sports, dancing, theatre, chess, photography, etc.)? Responses were dichotomized into participation in ECA's *yes* vs. *no*.

Five questions were asked to develop a score to assess each student's perception of the quality of ECAs at their school. The average score was computed and a higher score is indicative of a favorable environment for ECAs. Tertiles were used to categorize the student's perception into unfavorable, moderately favorable, and very favorable.

### School-level measures

The school economic deprivation score was developed by the Ministry of Education of Québec and is comprised of two indicators. First, the socio-economic index is made of the proportion of families with children whose mothers did not have a diploma, certificate or degree (which represents two thirds of the weight of the index) and the proportion of households whose parents were not employed during the reference week of the Canadian census (which is one third of the weight of the index). Second, the Low Income Cutoff (LICO) is defined as the income level that it is estimated that families spend 20% more than average on food, shelter and clothing. It provides information that is used to estimate the proportion of families whose income may be considered low, taking into account family size and area of residence (rural, small urban, large city). From these two measures, a score was developed. The lowest four deciles were categorized as *low economic deprivation*, three middle deciles were categorized as *moderate economic deprivation*, and the top three schools were categorized as *high economic deprivation*.

Using data from the Ministry of Education of Quebec, a school social fragmentation summary score was calculated using three school-level variables: proportion of young teachers, stability of teachers based on the teacher turnover rate, and proportion of students leaving school without a diploma. Proportion of young teachers (less than 5 years experience) is an indication of instability (i.e. the proportion of young teachers was inversely correlated with teacher stability). A school with a large proportion of young teachers is an indication of having teachers with short tenure. A Principal Component Analysis (PCA) was conducted and all three indicators loaded on the same factor (Cronbach alpha = 0.70). Using the refined regression method, a Social fragmentation score was determined (mean = 0, SD = 1.0). A higher social fragmentation score reflects greater social fragmentation of the school. The scores were categorized into tertile groups: low, moderate, and high.

To assess adolescents' perceptions of social cohesion, we used a thirty-seven item instrument [38], which was composed of seven separate subscales: Relationships between students, Relationships between students and teachers, Education, (i.e. Perception of the quality of the environment for learning), Security (i.e. perception of safety), Justice (i.e. students are treated fairly), Equity, the staff members (teachers, supervisors, administration team, etc.) treat all students the same way, and Membership or belonging. An overall social cohesion score was creating by adding each of the subscales to develop an overall score (Cronbach alpha = 0.92). A higher social cohesion score was indicative of a more positive perception of the school. A PCA of the social cohesion instrument indicated acceptable construct validity since all items loaded strongly on each of the theoretically predicted dimensions [38], [40].

The average school social cohesion score was determined for each school by aggregating student responses within each school. The range was 26.77–34.59; and mean score was 30.06 (SD = 1.67). We categorized the school social cohesion scores of the schools into low, moderate, and high based on the tertiles.

All three sets of indicators pertaining to school level economic deprivation, social fragmentation, and social cohesion were included in a principal components analysis to confirm that all indicators loaded onto their respective socioeconomic factor. As expected, all indicators loaded onto three socioeconomic factors, which is an indication that these three factors were orthogonal to each other.

### Analyses

For all analyses, multilevel modeling was used to account for the clustering within schools. At baseline, we used a two-level logistic regression model to investigate the cross-sectional and prospective association between school level socio-economic exposures and physically inactivity above and beyond confounders. At baseline, n = 14,924 (nested within 75 schools) students with complete data were included. Five schools were excluded due to missing Ministry of Education of Quebec data. For the longitudinal analysis, students, n = 6656 (nested within 69 schools) who reported their physical activity levels at follow-up were retained for analysis. A further six schools were removed due to loss to follow-up. We did not account for the two time points. Students who were inactive at baseline were excluded from the longitudinal analysis to determine the incidence of physical inactivity. However, we determined if similar findings were obtained when those who participated in no physical activity at baseline were included and we controlled for physical inactivity behavior at baseline. Since most 18 year olds were not followed, they were excluded from the analysis.

To investigate the association between the school level socio-economic exposures and physical inactivity at baseline and at follow-up, we fitted the following sequence of models, using a step-up approach [41]. First, a set of analysis involved estimating a school-level intercept-only model, in order to determine the 95% plausible value range of the degree of variability between schools in risk of physical inactivity. Also, the null model was used to determine the Intraclass Correlation (ICC), which indicates the proportion of the total variance that occurred between schools [42]. The next model introduced individual level demographics. A third model only included the school level variables, while the fourth model included both individual and school level covariates. Finally, a model that included participation in ECAs and perception of availability of ECAs at school were added. Sex and school cross-level interactions were also tested but findings were not significant (results not shown). Analyses were performed using SPSS (version 20.0) and HLM 6.04 (Hierarchical Linear Modeling, Scientific Software International, Chicago, IL).

### Sub-analyses

Since participation in ECA's and perception of the quality of ECA's were not measured within English schools, multiple logistic regression analyses were repeated that included these two student-level variables among the French schools only (Cross-sectional: n = 12,864; Longitudinal: n = 5,704).

## Results

The characteristics of the students at baseline and those who were followed are presented in Table 1. At baseline, there were slightly more females (54.8%), a majority were Canadian-born (84.9%) and were from a family with 2 or more parents (88.6%). The average individual social cohesion score was 29.7, SD = 5.4, and the range was 7.0–42.0.

**Table 1:** Characteristics of the cross-sectional sample at baseline.

	n = 14924		n = 6656		
	n	%	n	%	p-value
**Sex**					
Male	6746	45.2	2850	42.8	<0.01
Female	8178	54.8	3806	57.2	
**Family Status**					
2 or more parents	13218	88.6	5902	88.7	0.83
Single parent	1706	11.4	754	11.3	
**Immigrant Status**					
Canadian born	12663	84.9	5659	85.0	0.10
Aboriginal	174	1.2	76	1.1	
Non-European	1419	9.5	608	9.1	
European	668	4.5	313	4.7	
**Age**					
12 years	988	6.6	584	8.8	<0.01
13 years	2847	19.1	1544	23.2	
14 years	3248	21.8	1615	24.3	
15 years	3311	22.2	1785	26.8	
16 years	2808	19.5	1021	15.3	
17 years	1622	10.9	103	1.6	
**Physically Inactive**					
No	14215	95.2	6430	96.0	
Yes	709	4.8	266	4.0	
	Mean	SD	Mean	SD	
School Climate	30.0	5.4	30.3	5.2	

School Level Factors at baseline (n = 75)			School Level Factors at Follow-up (n = 69)		
	Mean	Range	Mean	Range	
Economic Deprivation	6.98	1–10	7.03	1–10	
Social Fragmentation	111.1	58.0–146.8	110.6	58.0–146.8	
Social Cohesion	29.97	26.77–34.59	30.05	27.23–34.59	

Among the schools (n = 76), school-level economic deprivation, social cohesion, and social fragmentation were not significantly correlated. Economic deprivation was negatively correlated with social cohesion (r = −.31, p<0.01) and positively associated with social fragmentation (r = 0.18, p = 0.14). Social cohesion and social fragmentation were negatively correlated (r = −0.19, p = 0.13).

The 95% plausible value range determined from the null multilevel model showed that the prevalence at baseline of children not participating in any physical activity ranged from 1.7% to 11.1% across schools. At follow-up, the 95% plausible range was determined to be 1.5% to 9.3% children reporting no physical activity across schools. The ICC's at baseline and at follow-up were determined to be 0.07, which indicates the proportion of the total variance that occurred between schools is 7%.

**Figure 1:**
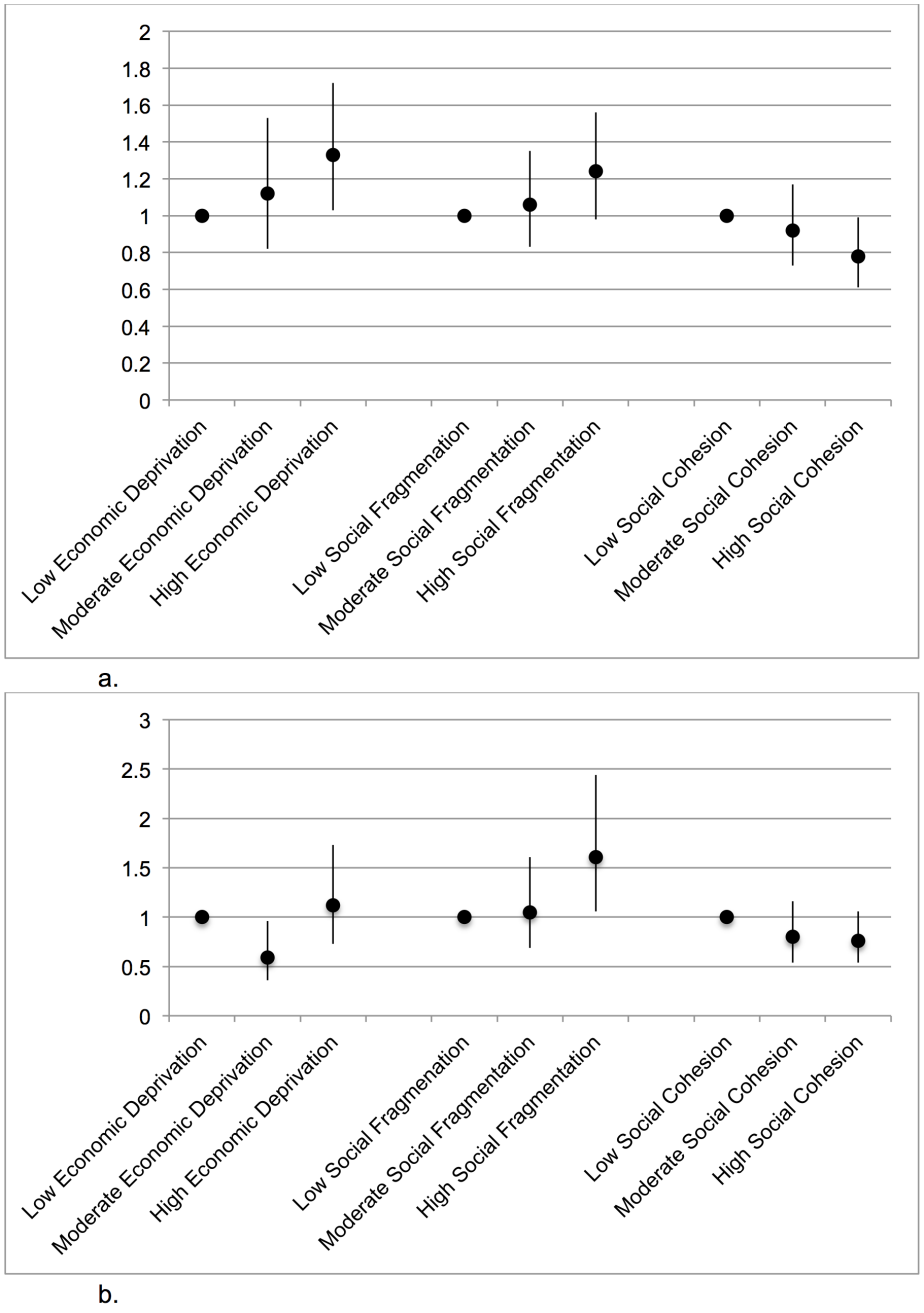
a. Cross-sectional multiple logistic regression findings examining the relationships between the school-level socio-economic factors and the odds for physical inactivity among students participating in the NANS Study 2006. b. Longitudinal multiple logistic regression findings examining the relationships between the school-level socio-economic factors and the odds for physical inactivity among students participating in the NANS Study 2006–2008.

At baseline, crude analysis indicated economic deprivation and social fragmentation were associated with an increased likelihood of physical inactivity in comparison to students attending low economic deprivation and social fragmentation schools (Table 2). Conversely, students attending schools with favorable social cohesion scores were less likely to be physically inactive. These relationships remained when controlling for confounders. High (OR = 1.33, 95% CI = 1.03, 1.72) economic deprivation and high (OR = 1.24, 95% CI = 0.98, 1.56) social fragmentation was associated with an increased likelihood of physical inactivity. Students attending high (OR = 0.78, 95% CI = 0.61, 0.99) socially cohesive schools were less likely to participate in no physical activity (Table 2; Figure 1a). Also, in comparison to the students attending French Language schools, those attending English schools were significantly more likely to be physically inactive at baseline (OR = 2.25, 95% CI = 1.74, 2.92).

**Table 2:** Cross-sectional associations between individual and school-level characteristics and physical inactivity.

	Model 1		Model 2		Model 3		Model 4**		Model 5	
	n = 14,924								n = 12,864	
	OR	95% CI	OR	95%CI	OR	95%CI	OR	95%CI	OR	95% CI
**Individual Characteristics**										
Intercept	0.05	0.04,0.06	0.07	0.04,0.12	0.03	0.02,0.05	0.05	0.03,0.10	0.07	0.03,0.17
**Sex** (ref: male)										
Female			1.31	1.12, 1.54			1.27	1.09,1.48	1.39	1.15,1.69
**Age** (ref: 12 year old)										
13 year old			1.24	0.87, 1.76			1.23	0.83,1.84	1.18	0.77,1.83
14 year old			1.35	0.89, 2.05			1.34	0.91,1.97	1.20	0.73,1.98
15 year old			1.43	1.02, 2.01			1.37	0.93,2.02	1.30	0.84,2.00
16 year old			1.70	1.24, 2.32			1.59	1.08,2.35	1.56	1.05,2.30
17 year old			1.88	1.29, 2.78			1.75	1.17,2.64	1.93	1.12,3.33
**Family Structure** (ref: >1 parent)										
Single Parent			1.17	0.96, 1.43			1.14	0.92,1.42	1.06	0.83,1.35
**Immigrant Status** (Canadian-born)										
Non-European			1.56	1.27, 1.92			1.34	1.03,1.73	1.46	1.10.1.94
European			1.49	1.09, 2.04			1.50	1.09,2.07	1.19	0.77,1.84
Aboriginal			0.92	0.44, 1.91			0.89	0.42,1.89	0.66	0.25,1.74
**SES Risk** (ref: high)										
Moderate			0.63	0.40, 0.99			0.72	0.60,0.86	0.65	0.39,1.10
Low			0.47	0.31, 0.73			0.70	0.56,0.88	0.49	0.30,0.80
**School Social Cohesion** (ref: low)										
Moderate			0.73	0.62, 0.85			0.71	0.60,0.85	0.87	0.71,1.07
High			0.59	0.49,0.70			0.58	0.48,0.71	0.77	0.60,0.99
**Participation in Extracurricular Activities** (ref: No)										
Yes									0.35	0.30,0.42
**Perception of Extracurricular Activities** (ref: weak)										
Moderate									0.86	0.59,1.26
Strong									0.62	0.42,0.93
**School Characteristics**										
**Language** (ref: French)										
English					2.31	1.81, 2.95	2.25	1.74, 2.92		
**Economic Deprivation** (ref: low)										
Moderate					1.15	0.87,1.52	1.12	0.82, 1.53	1.17	0.86,1.59
High					1.54	1.19, 1.98	1.33	1.03, 1.72	1.35	1.01,1.81
**Social Fragmentation** (ref: low)										
Moderate					1.09	0.84, 1.41	1.06	0.83, 1.35	1.26	0.92,1.73
High					1.35	1.05, 1.74	1.24	0.98, 1.56	1.38	1.03,1.84
**School Social Cohesion** (ref: low)										
Moderate					0.85	0.65, 1.12	0.92	0.73, 1.17	1.05	0.80,1.39
High					0.70	0.54, 0.89	0.78	0.61, 0.99	0.98	0.72,1.33

**The equation below is based on model 4 found in table 2.

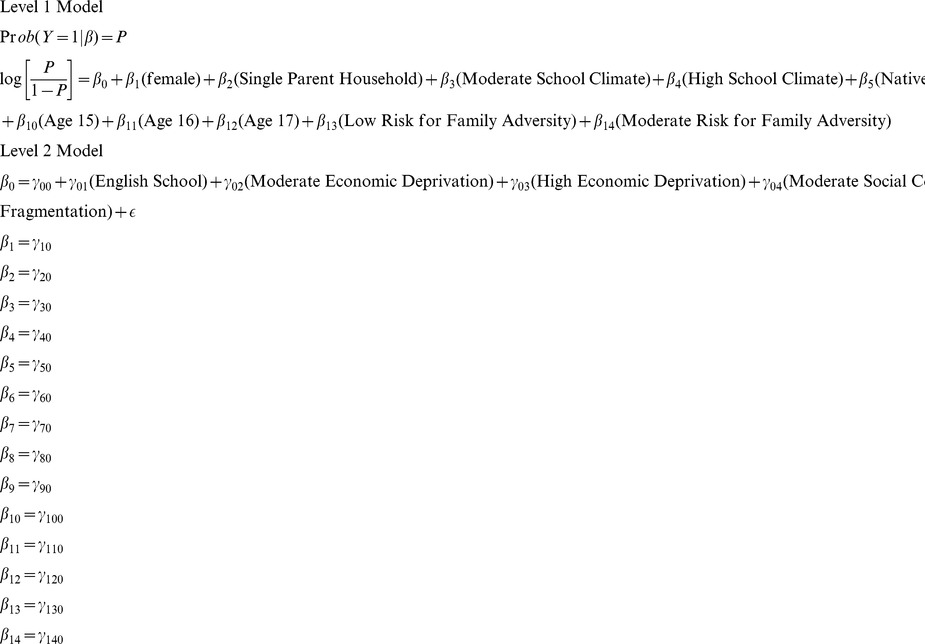

Of the students who were followed and who reported participating in any physical activity at baseline, n = 257, 3.9%, reported being physically inactive at follow-up. Students in high socially fragmented schools, in comparison to low socially fragmented schools were more likely to be physically inactive at follow-up (OR = 1.65, 95% CI = 1.09, 2.51) (Figure 1b). No association was found between school social cohesion and physical inactivity at follow-up.

Similar findings were obtained when those who participated in no physical activity at baseline were included in the analyses. In the fully adjusted model, those who participated in no physical activity at baseline were significantly more likely to participate in no physical activity at follow-up (OR = 9.49, 95% CI = 6.56, 13.73). In comparison to students attending low socially fragmented schools, students attending high socially fragmented schools were more likely to be physically inactive at follow-up (OR = 1.53, 95% CI = 1.05, 2.23). Also, in comparison to students attending French schools, those attending English school were significantly more likely to become physically inactive at follow-up. (OR = 2.20, 95% CI = 1.43,3.38).

### Sub analyses

When participation in ECA's and perception of the ECA's in the school environment were included in the model, results remained consistent. At baseline, high economic deprivation (OR = 1.35, 95% CI = 1.01, 1.81) and high social fragmentation (OR = 1.38, 95% CI = 1.03, 1.84) were associated with an increased likelihood of physical inactivity (Table 2). However, high social cohesion was no longer associated with a decreased likelihood of physical inactivity. Students in high socially fragmented schools, in comparison to low socially fragmented schools were more likely to be physically inactive at follow-up (OR = 1.62, 1.00, 2.62) (Table 3).

**Table 3:** Sub-group analysis of the association between individual and school level characteristics and physical inactivity among children who were active at baseline.

	Model 1		Model 2		Model 3		Model 4**		Model 5	
	n = 6,656								n = 5,704	
	OR	95% CI	OR	95%CI	OR	95%CI	OR	95%CI	OR	95% CI
**Individual Characteristics**										
Intercept	0.04	0.03,0.05	0.03	0.01,0.11	0.03	0.02,0.06	0.02	0.00, 0.10	0.09	0.02,0.49
**Sex** (ref: male)										
Female			1.03	0.79, 1.35			1.07	0.84,1.36	0.98	0.73,1.31
**Age** (ref: 12 year old)										
13 year old			1.85	0.91,3.79			1.79	0.94,3.40	2.08	0.91,4.79
14 year old			1.89	0.91, 3.93			1.92	1.02,3.62	1.76	0.75,4.14
15 year old			2.10	1.02, 4.35			2.15	1.15,4.04	2.18	0.86,5.51
16 year old			2.90	1.49, 5.64			2.99	1.57,5.70	2.75	1.27,5.97
17 year old			1.89	0.55, 6.47			2.47	0.84,7.22	2.26	0.63,8.17
**Family Structure** (ref: >1 parent)										
Single Parent			1.40	0.99, 1.97			1.68	1.21,2.33	1.47	1.00,2.15
**Immigrant Status** (Canadian-born)										
Non-European			0.80	0.53, 1.23			0.72	0.44,1.16	0.69	0.43,1.10
European			1.49	1.06, 2.11			1.37	0.82,2.30	1.32	0.72,2.39
Aboriginal			0.99	0.34, 2.86			0.86	0.27,2.71		
**SES Risk** (ref: high)										
Moderate			1.09	0.33, 3.62			1.05	0.80,1.38	1.07	0.32,3.58
Low			0.76	0.23, 2.54			0.80	0.56,1.16	0.76	0.23,2.52
**School Climate Perception** (ref: low)										
Moderate			0.56	0.42, 0.75			0.57	0.43,0.76	0.60	0.41,0.88
High			0.50	0.36, 0.70			0.52	0.38,0.70	0.52	0.34,0.80
**Participation in Extracurricular Activities** (ref: No)										
Yes									0.62	0.45,0.84
**Perception of Extracurricular Activities** (ref: unfavorable)										
Moderately favorable										
Very favorable										
**School Characteristics**										
**Language** (ref: French)										
English					2.05	1.40, 3.00	2.20	1.43,3.38		
**Economic Deprivation** (ref: low)										
Moderate					0.59	0.36, 0.96	0.54	0.34, 0.87	0.55	0.36,0.85
High					1.12	0.73, 1.73	1.07	0.70, 1.62	0.88	0.57,1.36
**Social Fragmentation** (ref: low)										
Moderate					1.05	0.69, 1.61	1.08	0.70,1.67	1.05	0.62,1.78
High					1.61	1.06, 2.44	1.65	1.09, 2.51	1.62	1.00,2.62
**Social Cohesion** (ref: low)										
Moderate					0.80	0.54, 1.16	0.87	0.59, 1.29	1.00	0.66,1.51
High					0.76	0.54, 1.06	0.89	0.62, 1.27	0.86	0.54,1.38

**The equation below is based on model 4 found in table 3.

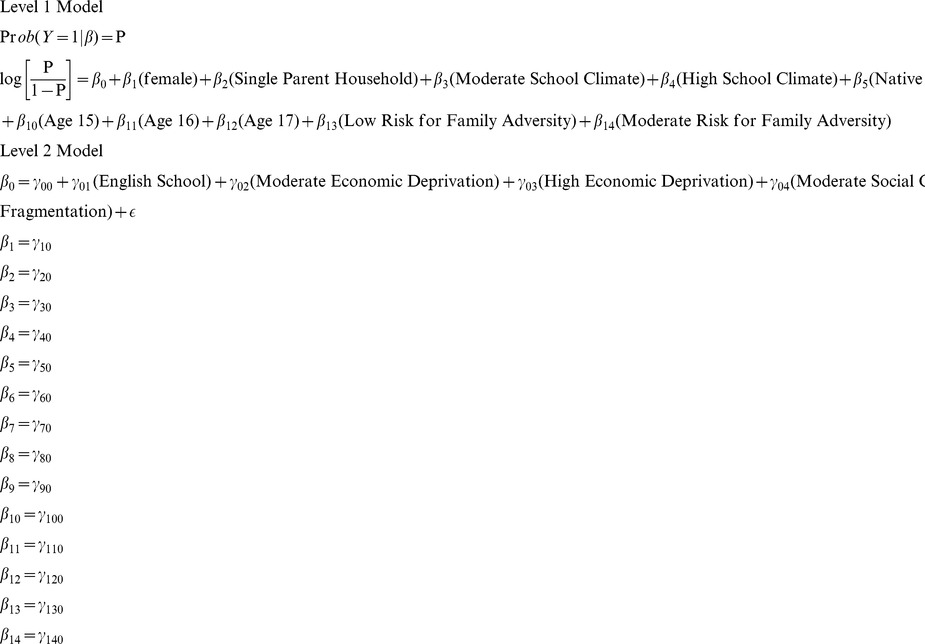

## Discussion

The objective of this investigation was to determine if the school-level characteristics, economic deprivation, social fragmentation, and school social cohesion, were associated with physical inactivity at baseline and at follow-up. Students attending schools that were categorized by high economic deprivation were more likely to be physically inactive at baseline while those attending high social cohesion schools were less likely to be physically inactive at baseline. Students attending schools with a high social fragmentation were more likely to be physically inactive at baseline or to become physically inactive at follow-up.

Previous studies have indicated that school resources devoted to physical activity tend to encourage physical activity among students. The presence and accessibility of sport fields and gymnasiums as well as equipment will promote physical activity [43], [44]. A supportive social environment may also be necessary to promote physical activity. Teachers, staff and peers may encourage or even discourage physical activity behavior. Our findings remained significant even when controlling for quality of ECAs offered at the school and participation in ECAs. This study adds to the literature because it illustrates how stability of the school environment, may influence physical activity.

Schools with high economic deprivation may have large proportions of students with limited access to resources needed for physical activity. Although the schools included in this study were among the most deprived in Quebec, those schools that are the most economically deprived may be more at risk for having limited funds and equipment. This may result in decreased opportunities for physical activity. Opportunities for physical activity, such as ECAs have been shown to be important for physical activity levels of adolescents [27], [45]. However, the perceived quality of the ECAs is just as important for physical activity.

Social fragmentation within a neighborhood has been investigated as an independent factor associated with physical activity [10], [34]. For example, social fragmentation was associated with a decreased likelihood of choosing walking as a form of exercise among mothers living in Quebec, Canada [34]. In unpublished work, we observed neighborhood social fragmentation as a risk factor for participating in no physical activity among Boston adolescents participating in the 2008 Boston Youth Study [46]. For this current investigation, we applied social fragmentation within a school setting as risk factor of participating in no physical activity.

Social fragmentation and physical inactivity may be mediated by students' perceptions of their environment, in this case, the school setting. Perceptions of safety, belonging, being surrounded by peers, have been shown to be associated with physical activity. Students who have more positive perceptions about their school may be more likely to participate in ECAs offered, such as team sports. For example, school connectedness has been defined as the extent to which students feel like they are part of the school [47], [48]. Previous research has indicated that feeling disconnected was associated with engaging in no vigorous physical activity among girls only [49]. A lower level of social fragmentation on the school level may lead to increased physical activity because increased stability might lead to a greater sense of connectedness to one's school. When students are surrounded by their peers who feel similarly about their school an amplification effect may occur.

Therefore, opportunities such as ECAs may not be enough to encourage physical activity behavior. Creating supportive and less socially fragmented environments may be needed to increase activity levels. Research has shown that schools that have teachers who are encouraging and supportive for physical activity are more likely to have students who are physically active [21], [22]. Decreasing student drop out rates and decreasing teacher turnover are some ways in which to increase the stability of the school environment. Successful interventions to decrease student drop out in the past have included creating safe, nonthreatening, learning environments, implementing mentoring programs, and creating small class sizes [50]. A desired outcome of decreasing dropout rates may be increased physical activity levels among students.

We also observed students attending English language schools were significantly more likely to be physically inactive at baseline and more likely to become physically inactive at follow-up in comparison to those attending French language schools. These findings are unlikely to be attributable to an influence of the language of instruction but rather due to other factors. For example, Bourhis & Foucher (2012) and Lamarre (2012) argue that since students attending English Schools in a predominantly French-Speaking province constitute a linguistic minority, low enrollment and subsequent defunding of these schools might have detrimentally affected the resources available for physical activity programs [51], [52]. That is, the low enrollment and defunding of English public schools in Quebec might have led to cut backs of extracurricular activities and resources needed for physical activity [51], [52].

Some limitations of our study include that physical activity was self-reported, and not validated by use of pedometers or accelerometers. Therefore, the question used might not be actually measuring physical activity. Although our assessment of physical activity was crude, we believe that the dichotomous form of ascertainment (“0 hours” vs. “1–12 or more hours” of physical activity) distinguished between those who were physically inactive from those who did any physical activity. A dichotomization of zero versus any physical activity is a more conservative estimate of physical inactivity. Misclassification of the outcome is therefore less likely compared to attempts to estimate daily MET-values. Furthermore, since physical activity levels decline during adolescents and our objective for this study is to describe the relationship between school-level social characteristics and participation in no physical activity, dichotomizing physical activity into zero versus any is practical.

Another limitation of our study is that there was a high attrition rate at follow-up. However, since the students lost to follow-up were more likely to be from deprived backgrounds, our results may have underestimated the true associations. Also, since physical activity levels were only measured at two time points during this longitudinal study, we were limited in our options for data analyses. By having more than 2 time points, we could have conducted growth curve analyses and therefore could have determined the effect of school level socio-economic factors on physical activity behavior throughout the secondary school years. Since the participating schools were mostly from low socioeconomic backgrounds, our ability to generalize findings to the general population is limited. Another limitation is the lack of racial or ethnic background and weight status information that may confound the relationship between school socio-economic factors and physical inactivity.

Other school-level factors that could potentially influence physical activity should be included in future analyses. Although perception of safety was included as a potential driver of social cohesion, objective measures, such as the rates of assaults or incidents within schools, should be used to account for safety. Physical disorder, such as the presence of vandalism in the school, should also be included. Also, another school level factor that could play a role in physical activity behavior is physical education classes offered in the curricula. Although there are certain requirements (i.e., 150 minutes per cycle of 9 days-approximately 83 minutes per week), schools may or may not adhere to requirements; in Quebec 69% of public schools conform to ministerial guidelines (Submitted article for peer review). For the purposes of this study, we do not know the adherence levels of participating schools. Future research should either take into account these classes of measure physical activity excluding physical education instruction.

In conclusion our study suggests that school level economic deprivation, social fragmentation, and perceptions of the school social cohesion might be related to an increased likelihood in participating in no physical activity above and beyond individual characteristics. School environments may be key areas to implement interventions to increase physical activity behavior. Offering opportunities for physical activity may not be sufficient. Creating a stable and supportive social environment may also be needed to facilitate participation in physical activity.
